# Effect of processing on the contents of amino acids and fatty acids, and glucose release from the starch of quinoa

**DOI:** 10.1002/fsn3.1775

**Published:** 2020-07-21

**Authors:** Ligen Wu, Anna Wang, Ruilin Shen, Lingbo Qu

**Affiliations:** ^1^ School of Food Science and Technology Henan University of Technology Zhengzhou China; ^2^ College of Food and Biological Engineering Zhengzhou University of Light Industry Zhengzhou China; ^3^ Henan Key Laboratory of Chemical Biology and Organic Chemistry Zhengzhou University Zhengzhou China

**Keywords:** amino acids, fatty acids, processing, quinoa

## Abstract

The effects of processing on the content of amino acids and fatty acids and the release of glucose from quinoa grains were evaluated in this paper. The processes included dehulling, boiling, extrusion, heating under pressure, and baking (infrared heating). The retention rate (AR) of essential amino acids and fatty acids of dehulled and boiled quinoa was 100%. The oil content of the extruded quinoa samples of two varieties was 47.71% and 39.75% lower than the corresponding raw quinoa samples. Baking and heating under pressure had different effects on the essential amino acid content, fatty acid content, and hydrolysis rate of quinoa starch. The results indicated the different cooking methods affect the essential amino acid content, fatty acid composition, release of glucose, and nutritional quality of quinoa, and moderate processing should be adopted to fully utilize the essential amino acids, fatty acids, and starch in quinoa.

## Graphical abstract

Thermal treatment has different effects on the essential amino acid content, fatty acid content, and the hydrolysis rate of quinoa starch. The proper processing methods for quinoa products to satisfy the nutritional demand of consumers are important. The results of this paper may help commercial or production industries easily choose the appropriate processing method to meet the consumer's request for nutrition in quinoa grain, effectively increase the value of quinoa products, and enhance its industrial profits.

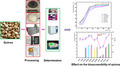

## 1 INTRODUCTION

Quinoa is an excellent source of macronutrients, especially proteins, with a high content of essential amino acids; the nutritional properties of quinoa are unique since it contains all essential amino acids, minerals (Nowak, Du, & Charrondière, 2016; Stikic et al., 2012), and vitamins (B6, folate, riboflavin, and niacin; Abugoch, 2009), which is different than common cereals (FAO, 2013). In addition, quinoa contains phenolic compounds (Dini, Tenore, & Dini, 2010). As a result, its popularity and cultivation area are expanding rapidly in many countries. In China, quinoa planting began in 1988 in Tibet, and it was introduced into Gansu Province, Shanxi Province, Qinghai Province, Hebei Province, Inner Mongolia Autonomous Region, and other places, and some local varieties with better adaptability have been selected, such as Mengli No. 1 and Qingli No. 2, since 2013.

Many data can be found on the nutrients, bioactive compounds, and antinutrients of quinoa in research reports and databases from the USDA (U.S. Department of Agriculture, 2013), Bolivia (Instituto Nacional de Laboratorios de Salud (INLASA), 2005), Peru (Centro nacional de alimentación y nutrición instituto nacional de salud, 2009), and ASEAN (Institute of Nutrition, 2014). The nutrient contents of quinoa varied considerably, where the highest value could be double or triple the amount of the lowest value (Kozioł, 1992; Nowak et al., 2016; Ruales & Nair, 1992; Vega‐Galvez et al., 2010). The differences are possibly due to the different varieties, cultivars, analytical methods, and environmental condition factors (Greenfield & Southgate, 2003; Toledo & Burlingame, 2006). More high‐quality analytical data on the nutritional composition of quinoa under different conditions, as well as for different varieties, are necessary (Nowak et al., 2016). Research results on the nutritional content of quinoa in China will help to enhance the awareness and utilization of different varieties of quinoa (Nowak et al., 2016).

Quinoa grains may be used in the production of food and be consumed similarly to cooked rice, used in porridge, baked goods, and extrusion products, or ground into flour (Vega‐Galvez et al., 2010). It is known that the type of processing used on food can affect the nutrient composition in various ways, including the bioactive compounds and antioxidant capacity; food processing may exert little or no change, or a reduction or maintenance of these properties (Chan et al., 2009; Nickel, Spanier, Botelho, Gularte, & Helbig, 2016). Heating treatments, such as cooking, can affect the nutritional components and contribute to protein oxidation, tryptophan degradation, and protein carbonylation (Santé‐Lhoutellier, Astruc, Marinova, Greve, & Gatellier, 2008; Soladoye, Juárez, Aalhus, Shand, & Estévez, 2015). Thermal processing, such as extrusion and roasting, on quinoa flour can result in degradation of saponin molecules (Brady, Ho, Rosen, Sang, & Karwe, 2007). Different drying temperatures may have different effects on the content of phenolic compounds and carotenoids in quinoa seeds (*Chenopodium quinoa*), and the concentration of phenolic compounds and carotenoids was shown to increase steadily with the increasing drying temperature (Multari, Marsol‐Vall, Keskitalo, Yang, & Suomela, 2018).

Quinoa represents a main protein source in diets, and quinoa constitutes a richer source of amino acids than rice and could be an alternative to rice in gluten‐free diets. The high content of Lys in quinoa reinforces its importance as a grain and highlights quinoa as the best source of amino acids (AAs). Studies on the amino acid bioavailability and bioaccessibility in food products are ongoing (Mota et al., 2016). The malting process was shown a method that produced a great effect than boiling and steaming on the amino acid content (Motta et al., 2019). However, food composition data are often only reported for uncooked forms of foods (US Department of Agriculture, 2011), and there are few data on the nutrient composition in cooked foods (Motta et al., 2019), especially regarding different cooking methods. However, little is known about the effects of heating under pressure, boiling, baking, and extruding on the amino acid and fatty acids (FA) composition of quinoa. Thus, it is important to evaluate the effect of these different processing types to which quinoa grains are subjected, to obtain more knowledge on nutrient retain after processing; this will be useful to determine the effect of cooking methods on the nutrient composition of foods, and it will help consumers select the suitable quinoa processing method by estimation of the nutrient intake. Therefore, this study aimed to evaluate the effects of five processing treatment, as dehulling, boiling, baking, extrusion, and heating under pressure on the contents of amino acids, fatty acids, and the release of glucose from quinoa grains, and to facilitate the informed decision‐making of consumers, policymakers, and food industries concerning the use of quinoa.

## 2 MATERIALS AND METHODS

### 2.1 Reagents

Porcine pancreas α‐amylase (E.C. 3.2.1.1) (A6255 type I‐A approx. 1,050 units/mg protein in saline solution, 29 mg proteins/ml), pepsin from gastric porcine mucosa (P7000), and protease type XIV isolated from *Streptomyces griseus* (P5147) were purchased from Sigma‐Aldrich. Amyloglucosidase from *Aspergillus niger* (EC 3.2.1.3., 3,300 units/ml on soluble starch, stabilized liquid in 50% (v/v) glycerol, 0.02% sodium azide) and isoamylase from *Pseudomonas* sp. (EC 3.2.1.68, 500 U/ml in ammonium sulfate suspension, 0.02% sodium azide) and a glucose assay kit based on enzymatic method (Mutarotase‐GOD) were purchased from Megazyme International Ireland Ltd. Standards of fatty acid methyl esters (FAMEs), methanol, KOH, methane sulfonic acid, tritridecanoin, sodium methoxide, and disodium hydrogen citrate sesquihydrate were obtained commercially from Sigma‐Aldrich. The other chemicals were reagent grade and obtained from Sigma‐Aldrich.

### 2.2 Quinoa grains

The two cultivated varieties of quinoa grains (Q1 and Q2) were grown in Qinghai Province of China and stored in refrigerated multilayer vacuum bags.

### 2.3 Processing of quinoa grains

The quinoa grains were analyzed raw (Q1‐Q and Q2‐Q) and later subjected to five different types of processing, which are described as follows. The quinoa grains were milled using a laboratory scale abrasive mill (JNM‐III abrasive miller; Chengdu Sweet Technology Co. Ltd.) and then sieved (sieves with a size of 710 μm). The dehulled grain was collected over the 710‐μm sieve and weighed for a dehulling rate of 8.6% (Q1‐MM, Q2‐MM).

All processing types had been previously tested and standardized in the laboratory and were performed in triplicate.

#### 2.3.1 Extrusion and heating under pressure process

Extrusion was performed according to Kowalski, Medina‐Meza, Thapa, Murphy, and Ganjyal (2016). The extrusion process utilized an 18‐mm corotating twin screw extruder (ZSE 18 HP, American Leistritz Extruder Corp). The extruder consisted of four independent temperature zones plus a feed zone. The overall length of the extruder barrel was 504 mm, giving a length‐to‐diameter (L/D) ratio of 28:1. The screws used had a modulated screw profile that was varied during preliminary trials for optimization. A cylindrical die with a diameter of 3 mm was used for all extrusion trials. The temperature profile of the extruder had the feed zone set at 22°C with the following zones set at 45, 70, and 100°C, and the last zone was varied at 120, 140, and 160°C. The feed rate of the flour was fixed at 3.0 kg/hr using a twin screw gravimetric feeder. The screw speed of the extruder was varied from 300 to 500 rpm. Extrudates were collected under steady‐state conditions of pressure, torque, and temperature (after 5 min of steady‐state processing) and dried in a convection oven at 45°C for 18 hr yielding an average moisture content of 4%–6% (w.b.). The dried extrudates (recorded as Q1‐JY and Q2‐JY) were then stored in airtight plastic bags at 4°C for further analysis.

Heating under pressure was carried out under the condition of dry and hot compression by a special device designed in house. Quinoa grains were heated under 0.8 MPa pressure for 3–5 min. After cooling, the treated samples were crushed in an ultrafine grinder. The samples were recorded as Q1‐RY and Q2‐RY.

#### 2.3.2 Boiling and baking process

Boiling was performed according to Motta et al. (2019). Briefly, boiling was performed at 100°C for 15 min. For each 50 g of raw sample, 250 g of ultrapure water was added. After boiling, the samples were allowed to cool for 30 min. Boiled samples (recorded as Q1‐ZZ and Q2‐ZZ) were freeze‐dried before analysis.

The baking process was performed at 180°C for 25 min, and the baked quinoa samples (recorded as Q1‐HH and Q2‐HH) were cooled to 25°C.

The samples were then ground in a high‐speed grinder and stored separately in aluminum foil vacuum bags at 4°C until use.

#### 2.3.3 Standard AA analysis

Aqueous hydrochloric acid (HCl) 0.1 N was used to prepare a stock solution of D‐norvaline at a concentration of 2.5 mM to add to a standard solution and a concentration of 25 mM to add to samples. Additionally, a solution of 6 N HCl containing 0.5% phenol was used to dilute the samples and hydrolysis was completed in an oven for 10 hr at 110°C. The extracts were neutralized with 1 ml sodium hydroxide (6 N), and deionized water was used to bring the total volume to 10 ml. The hydrolyzates were filtered through filter paper before derivatization, which was performed by adding 80 µl of buffer, 10 µl of sample, and 20 µl of reconstituted derivatization reagent in a chromatographic vial. The reaction mixture was vortexed and immediately heated to a constant temperature of 55°C for 10 min. A Waters‐AccQ Fluor Reagent Kit, containing 6‐aminoquinolyl‐N‐hydroxysuccinimidyl carbamate as a derivatizing compound, was used with sample dilution buffer and eluents A (5% AccQ‐Tag Ultra Eluent in deionized water) and B (AccQ‐Tag Ultra Eluent, Waters Corporation) as mobile phase. The following gradient conditions used were as follows: 0–0.54 min, 99.9% A–0.1% B; 5.74 min, 90.9% A–9.1% B; 7.74 min, 78.8% A–21.2% B; 8.04 min, 40.4% A–59.6% B; 8.70–10 min, 99.9% A–0.1% B. The total chromatographic run time was 10 min (Mota et al., 2016). Tryptophan was determined using alkaline hydrolysis (AOAC International, 1995).

Working standard solutions were prepared from an amino acid hydrolyzate standard provided by Waters.

#### 2.3.4 Amino acid score

The amino acid score (AAS) of the EAAs was calculated according to the WHO (2007) report, applying the following equation:

(1) AAS=mgofaminoacidin1gtestprotein/mgofaminoacidinrequirementpattern

The amount of an amino acid in the requirement pattern was that for adults according to the FAO/WHO pattern.

### 2.4 Fatty acid analysis

Lipids extracted from the quinoa samples were quantified according to an AOAC method (AOAC, 1990; Hara & Radin, 1978), while transesterification of FAs was performed by direct transesterification with methanolic sodium methoxide with addition of the internal standard solution (tritridecanoin) to the oil extracted from the quinoa samples to prepare fatty acids methyl esters (FAMEs) as described by Golay and Moulin (2016), Christie (1982), and Chouinard, Corneau, Sæbø, and Bauman (1999). The FAs were analyzed as their methyl esters by an Agilent 7890 gas chromatograph (Agilent Technologies, Inc.), equipped with an HP‐8 capillary column (100 m × 250 µm × 0.2 µm). The injector and detector ports were set at 250 and 300°C, respectively. The oven temperature program was initially set at 170°C for the first min, increased at a rate of 4°C/min to 220°C, increased at a rate of 1°C/min to 240°C, and held for 32.5 min. The carrier gas was helium. One microliter of FAME sample was injected with a 1:50 split ratio.

### 2.5 Determination of retention

The apparent retention factor (AR) was calculated on a moisture‐free basis (dry weight basis), according to the US Department of Agriculture (2007).

(2) $$\text{AR}\;\left(\%\right)\;=\;\left[A\;\left(\text{dry}\;\text{weight}\;\text{basis}\right)/B\;\left(\text{dry}\;\text{weight}\;\text{basis}\right)\right]\;\times \;100$$

where A is the nutrient content (g) of cooked quinoa and B is the nutrient content (g) of raw quinoa.

### 2.6 Enzymatic hydrolysis of quinoa flour

Quinoa flour containing 100 mg starch (dry basis) was weighed into 50‐ml polypropylene copolymer tubes and mixed thoroughly with 3.5 ml of distilled water. The digestion procedure was modified from Srichuwong et al. (2017). The quinoa flour suspension was kept at 37°C for 5 min and combined with 1.5 ml of pepsin–HCl solution (1.35% w/w pepsin, 0.05 M HCl, pH 2.0), and the mixture was incubated at 37°C for 30 min on a magnetic stirrer. The pH was brought up to 6.0 by adding 3.0 ml pH 6.4 maleate buffer (0.1 M, 10 mM CaCl_2_). To initiate starch digestion, 2 ml of enzyme solution (0.1 M maleate buffer pH 6.0, 10 mM CaCl_2_) containing 110 units of porcine pancreas α‐amylase and 33 units of amyloglucosidase was added. Starch digestion was performed at 37°C with a magnetic stirrer. Aliquots of 0.5 ml were taken at selected time intervals and immediately added to 1.5 ml of a cold ethanol solution (90% v/v). The mixture was kept in an ice bath for 10 min and then centrifuged (3,000 *g*, 10 min) to separate the supernatant. The collected supernatant was analyzed for glucose content using the assay kit. The glucose released from starch hydrolysis was obtained by subtracting the resulting glucose obtained from the tested sample with values obtained from blanks (a tube without sample and a tube without enzyme). The resulting glucose was multiplied by a factor of 0.9 to convert the glucose concentration into the starch and reported as a percentage of total starch content (dry basis).

## 3 RESULTS AND DISCUSSION

### 3.1 The effect of processing on the nonessential amino acids in quinoa

Glutamic acid was the highest amino acid in all samples (Table 1), and the contents of glutamic acid in the baked quinoa samples, Q1‐HH and Q2‐HH, were 219.56 mg/g protein and 245.23 mg/g protein, respectively, which were the higher than those of the other samples, while those in the raw quinoa samples, Q1‐Q and Q2‐Q, were 198.57 mg/g protein and 202.18 mg/g protein, respectively. The contents of glutamic acid were the highest in buckwheat, oat (Klose, Schehl, & Arendt, 2009), amaranth, and broad bean (Hejdysz, Kaczmarek, & Rutkowski, 2016). The contents of methionine and cysteine in the quinoa samples were the lowest, <19 mg/g protein, which was consistent with that in quinoa reported by Nowak et al. (2016) and in oats reported by Klose et al. (2009). The content of arginine in quinoa samples was 107.13–140.59 mg/g protein, which was close to that (109.60–129.20 mg/g protein) in other quinoa samples (Motta et al., 2019), less than that in buckwheat, and higher than that in rice, flour and sorghum (Mokrane et al., 2010). The range of tryptophan content was 88.28–122.59 mg/g protein, much higher than that in rice (0.5 mg/g protein) and wheat flour (1.24 mg/g protein) (Mokrane et al., 2010).

**TABLE 1:** The content of nonessential amino acids in quinoa samples/mg/g protein

	Asp	Ser	Glu	Gly	Ala	His	Arg	Pro	Trp
Q1‐Q	114.52 ± 2.47^g^	53.54 ± 1.38^gh^	198.56 ± 3.54^d^	77.24 ± 2.54^ef^	65.35 ± 1.32^d^	36.59 ± 1.22^b^	135.24 ± 2.45^h^	58.04 ± 3.9^e^	91.16 ± 3.30^c^
Q1‐MM	114.54 ± 0.51^g^	52.85 ± 0.80^g^	197.71 ± 1.70^cd^	73.34 ± 0.70^c^	63.89 ± 0.29^c^	44.23 ± 2.20^c^	131.41 ± 3.39^f^	61.52 ± 1.31^g^	93.07 ± 1.39^d^
Q1‐HH	124.22 ± 1.40^h^	59.68 ± 1.19^j^	219.56 ± 0.50^f^	84.36 ± 1.39^h^	72.88 ± 2.40^f^	53.86 ± 2.30^i^	141.18 ± 1.50^i^	66.14 ± 2.29^i^	71.40 ± 0.86^a^
Q1‐JY	111.93 ± 3.51^f^	52.65 ± 2.30^g^	195.83 ± 1.50^c^	74.35 ± 0.89^c^	63.49 ± 1.45^c^	47.24 ± 1.40^fg^	119.97 ± 2.70^b^	57.14 ± 1.39^e^	88.27 ± 1.39^b^
Q1‐ZZ	98.66 ± 1.40^a^	47.59 ± 1.49^e^	170.74 ± 2.70^a^	65.01 ± 2.50^a^	55.44 ± 2.35^a^	33.05 ± 0.95^a^	107.14 ± 1.49^a^	47.11 ± 2.40^d^	88.47 ± 3.21^b^
Q1‐RY	109.17 ± 2.41^e^	55.28 ± 1.30^f^	189.08 ± 1.51^b^	73.43 ± 1.35^c^	62.75 ± 0.40^c^	45.00 ± 0.89^cd^	125.39 ± 0.53^d^	60.12 ± 3.40^f^	101.17 ± 1.49^f^
Q2‐Q	115.75 ± 1.70^g^	54.39 ± 0.99^h^	202.18 ± 0.49^e^	79.87 ± 1.30^g^	68.87 ± 2.29^e^	49.52 ± 0.69^h^	133.37 ± 2.35^g^	64.79 ± 1.28^h^	106.19 ± 2.30^h^
Q2‐MM	102.26 ± 2.19^bc^	31.48 ± 2.20^b^	237.02 ± 2.40^i^	73.39 ± 3.34^c^	66.49 ± 0.79^d^	46.38 ± 1.29^ef^	128.87 ± 4.39^e^	44.97 ± 0.98^b^	122.58 ± 4.28^j^
Q2‐HH	106.92 ± 1.90^d^	27.58 ± 1.20^a^	245.23 ± 1.49^j^	78.44 ± 1.49^fg^	59.07 ± 1.49^b^	46.08 ± 0.49^de^	130.84 ± 1.30^f^	45.97 ± 1.29^bc^	113.13 ± 3.39^i^
Q2‐JY	104.01 ± 2.51^c^	45.69 ± 1.30^d^	237.22 ± 3.39^i^	74.48 ± 1.29^cd^	67.74 ± 2.22^e^	45.08 ± 2.29^cd^	123.56 ± 1.41^c^	46.57 ± 0.65^cd^	104.77 ± 0.93^g^
Q2‐ZZ	101.86 ± 3.59^b^	42.61 ± 1.39^c^	226.09 ± 5.59^h^	71.23 ± 1.59^b^	62.84 ± 1.21^c^	46.95 ± 1.20^efg^	108.26 ± 0.49^a^	38.01 ± 0.59^a^	90.39 ± 2.39^c^
Q2‐RY	110.00 ± 3.49^e^	55.87 ± 2.40^i^	198.36 ± 3.60^d^	75.84 ± 0.97^de^	63.24 ± 2.39^c^	47.78 ± 2.30^g^	121.91 ± 2.69^c^	60.51 ± 1.40^fg^	98.15 ± 1.49^e^

Note

Mean ± *SD* (standard deviation); means in the same column with different superscripts are significantly different (*p* < .01) (*N* = 3).

The contents of serine, glycine, alanine, and arginine decreased significantly (*p* < .01), and the content of tryptophan increased significantly (*p* < .01) in the dehulled samples (Q1‐MM and Q2‐MM) compared to those in the raw samples. The contents of aspartic acid, serine, glycine, alanine, arginine, proline, and tryptophan in the extruded quinoa samples (*p* < .01) significantly decreased, and the contents of glutamic acid and histidine in the extruded quinoa samples were significantly different from those in raw quinoa (*p* < .01). Heating under pressure significantly decreased the contents of aspartic acid, glutamic acid, glycine, alanine, arginine, and proline, and significantly increased (*p* < .01) the content of serine, and the contents of histidine and tryptophan in the heated processed samples were significantly different (*p* < .01) from those in raw quinoa. Compared to the raw quinoa, the content of nonessential amino acids in the processed quinoa samples decreased significantly (*p* < .01), which was consistent with the results reported by Motta et al. (2019) and Słupski (2010).

### 3.2 The effect of processing on the essential amino acids

The quinoa samples showed a favorable amino acid balance with the proportion of 43% of essential amino acids, which makes quinoa nutritionally suitable. The essential amino acid content in the dehulled quinoa (Q1‐MM and Q2‐MM) (Table 2) was higher than that in the responding raw quinoa (Q1‐Q and Q2‐‐Q) (*p* < .01). The contents of cysteine, methionine, lysine, threonine, isoleucine, leucine, tyrosine + phenylalanine, and lysine were 8.59% and 5.28%, 1.73% and 3.82%, 3.64% and 5.41%, 5.03% and 6.54%, 3.96% and 4.11%, 1.59% and 3.58% higher in Q1‐MM and Q2‐MM, respectively, than in the corresponding raw quinoa samples. This was because the essential amino acid content of the pericarp may be less than that of embryo and endosperm in quinoa; in fact, previous research showed that the content of protein was different in the pericarp, embryo, and endosperm of quinoa grains, and the protein in the embryo accounted for 57% of the total protein in quinoa (Ando et al., 2002; Nowak et al., 2016).

**TABLE 2:** The content of essential amino acids in quinoa samples/mg/g protein

	Cys + Met	Val	Thr	Ile	Leu	Tyr + Phe	Lys
Q1‐Q	17.35 ± 0.39^b^	42.68 ± 0.41^f^	31.04 ± 0.39^cd^	36.96 ± 0.28^de^	60.53 ± 1.16^fg^	80.64 ± 2.18^h^	57.68 ± 2.09^g^
Q1‐MM	18.83 ± 0.78^de^	43.43 ± 0.28^f^	32.17 ± 1.28^ef^	38.83 ± 1.19^g^	62.93 ± 0.34^h^	81.92 ± 1.19^i^	59.06 ± 2.28^h^
Q1‐HH	18.41 ± 0.26^cde^	43.08 ± 0.38^f^	33.19 ± 0.32^f^	38.98 ± 0.32^g^	63.45 ± 0.31^h^	79.95 ± 3.29^h^	55.29 ± 1.23^e^
Q1‐JY	17.17 ± 0.51^b^	38.57 ± 0.40^bc^	29.38 ± 1.14^b^	33.74 ± 1.42^bc^	55.07 ± 1.11^c^	72.26 ± 1.19^a^	51.34 ± 0.69^b^
Q1‐ZZ	17.33 ± 0.28^b^	39.07 ± 1.20^cd^	30.06 ± 0.14^bc^	36.97 ± 0.28^de^	55.86 ± 2.30^cd^	73.55 ± 1.19^d^	52.86 ± 0.25^d^
Q1‐RY	18.41 ± 1.30^cde^	37.87 ± 0.33^b^	30.43 ± 0.22^c^	32.97 ± 0.31^b^	52.77 ± 1.18^b^	70.25 ± 0.87^c^	47.72 ± 1.17^a^
Q2‐Q	17.44 ± 0.41^bc^	40.04 ± 0.39^de^	29.18 ± 0.28^b^	34.54 ± 1.17^c^	56.18 ± 0.18^d^	73.68 ± 1.28^d^	55.34 ± 2.16^e^
Q2‐MM	18.36 ± 0.29^cde^	41.56 ± 0.25^cde^	31.76 ± 1.19^de^	37.80 ± 2.03^f^	59.49 ± 0.40^e^	77.12 ± 1.35^f^	56.32 ± 1.30^f^
Q2‐HH	18.74 ± 1.09^de^	40.50 ± 0.29^e^	31.34 ± 0.19^de^	37.74 ± 1.02^ef^	61.47 ± 2.17^g^	70.20 ± 1.20^h^	54.47 ± 2.20^e^
Q2‐JY	18.98 ± 0.21^e^	36.66 ± 0.19^a^	27.74 ± 1.09^a^	31.52 ± 1.73^a^	53.04 ± 1.20^b^	68.69 ± 2.20^g^	51.05 ± 1.20^b^
Q2‐ZZ	18.80 ± 1.20^de^	37.93 ± 1.20^b^	29.16 ± 0.50^b^	34.68 ± 1.95^c^	55.14 ± 0.50^c^	73.13 ± 1.20^d^	53.47 ± 2.17^d^
Q2‐RY	18.61 ± 0.34^de^	36.13 ± 1.37^a^	29.36 ± 0.28^b^	31.96 ± 1.23^a^	51.58 ± 1.32^a^	67.18 ± 1.31_b_	47.65 ± 1.27^a^

Note

Mean ± *SD* (standard deviation); means in the same column with different superscripts are significantly different (*p* < .01) (*N* = 3).

The contents of cysteine, valine, leucine, and isoleucine in Q1‐HH and Q2‐HH were higher than those in the corresponding raw quinoa samples (Q1‐Q and Q2‐Q), while the contents of tyrosine + phenylalanine and lysine were less than those in the raw quinoa samples. The content of each essential amino acid in the two varieties Q1 and Q2 increased or decreased simultaneously and was affected by the baking treatment. The contents of leucine and isoleucine were 6.93% and 5.46%, 4.82% and 7.4%, and 9.26% and 10.2% higher, respectively, in samples Q1‐HH and Q2‐HH than the corresponding raw quinoa samples. The essential amino acids in boiled quinoa (Q1‐ZZ and Q2‐ZZ) were lower than those in Q1‐Q and Q2‐Q. The content of valine, leucine, and lysine were 8.46% and 7.72%, 8.36% and 4.12%, and 1.78% and 3.38% lower in Q1‐ZZ and Q2‐ZZ, respectively, than the corresponding raw quinoa samples.

The contents of all essential amino acids in Q1‐JY and Q2‐JY, the extruded samples, were less than those in Q1‐Q and Q2‐Q. The contents of valine, threonine, isoleucine, leucine, tyrosine + phenylalanine, and lysine in samples Q1‐JY and Q2‐JY were 9.63%, 6.51%, 8.71%, 9.02%, 10.39%, and 10.99% and 8.44%, 4.93%, 8.74%, 5.59%, 6.77%, and 6.99% lower than in Q1‐Q and Q2‐Q, respectively.

The essential amino acid contents of Q1‐RY and Q2‐RY were less than those of the control samples Q1‐Q and Q2‐Q, and the contents of valine, isoleucine, leucine, tyrosine + phenylalanine, and lysine in Q1‐JY and Q2‐JY were 11.27%, 10.8%, 12.82%, 8.79%, and 17.27% and 9.77%, 7.47%, 8.19%, 8.82%, and 13.90% lower than in the control samples, respectively, due to the heated under pressure treatment, and the loss of lysine in Q1‐JY and Q2‐JY was notably much larger than that in the extruded, baked, boiled samples.

### 3.3 The effect of processing on the essential amino acid score of quinoa

The essential amino acid scores of the quinoa samples are shown in Table 3, the aromatic amino acids (AAAs‐phenylalanine and tyrosine) of the raw quinoa samples presented the highest AAS values. This finding was in agreement with the results of Motta et al. (2019), Nowak et al. (2016) and Srichuwong et al. (2017).

**TABLE 3:** The essential amino acids scores in quinoa samples

	Cys + Met	Val	Thr	Ile	Leu	Tyr + Phe	Lys	AR of EAA
Q1‐Q	0.49 ± 0.11^b^	0.85 ± 0.08^f^	0.77 ± 0.09^cd^	0.92 ± 0.07^de^	0.86 ± 0.12^fg^	1.34 ± 0.03^h^	1.05 ± 0.07^g^	/
Q1‐MM	0.56 ± 0.08^de^	0.87 ± 0.15^f^	0.80 ± 0.07^ef^	0.97 ± 0.05^g^	0.89 ± 0.01^h^	1.36 ± 0.13^i^	1.07 ± 0.02^h^	1.0
Q1‐HH	0.54 ± 0.05^bcde^	0.86 ± 0.17^f^	0.83 ± 0.08^f^	0.97 ± 0.08^g^	0.91 ± 0.15^h^	1.33 ± 0.09^h^	1.04 ± 0.04^g^	1.0
Q1‐JY	0.51 ± 0.11^cd^	0.77 ± 0.08^bc^	0.74 ± 0.07^b^	0.84 ± 0.10^bc^	0.78 ± 0.12^c^	1.05 ± 0.13^a^	0.92 ± 0.12^b^	0.9
Q1‐ZZ	0.53 ± 0.11^bcde^	0.78 ± 0.14^cd^	0.75 ± 0.04^bc^	0.92 ± 0.07^de^	0.79 ± 0.14^cd^	1.23 ± 0.23^d^	0.96 ± 0.05^d^	1.0
Q1‐RY	0.54 ± 0.21^bcde^	0.75 ± 0.17^b^	0.68 ± 0.06^a^	0.82 ± 0.07^b^	0.75 ± 0.13^b^	1.17 ± 0.14^c^	0.87 ± 0.17^a^	0.9
Q2‐Q	0.49 ± 0.07^bc^	0.80 ± 0.08^de^	0.73 ± 0.07^b^	0.86 ± 0.14^c^	0.80 ± 0.13^d^	1.23 ± 0.15^d^	1.01 ± 0.13^e^	/
Q2‐MM	0.52 ± 0.06^bcde^	0.79 ± 0.05^cde^	0.79 ± 0.11^de^	0.94 ± 0.08^f^	0.85 ± 0.11^e^	1.29 ± 0.08^f^	1.02 ± 0.15^f^	1.0
Q2‐HH	0.54 ± 0.04^de^	0.81 ± 0.06^e^	0.78 ± 0.15^de^	0.94 ± 0.07^ef^	0.88 ± 0.12^g^	1.24 ± 0.23^d^	1.01 ± 0.14^e^	1.0
Q2‐JY	0.54 ± 0.06^e^	0.79 ± 0.04^cde^	0.79 ± 0.12^de^	0.94 ± 0.11^ef^	0.77 ± 0.13^g^	1.31 ± 0.13^g^	0.93 ± 0.13^c^	0.9
Q2‐ZZ	0.49 ± 0.09^bc^	0.76 ± 0.04^b^	0.75 ± 0.12^b^	0.92 ± 0.11^d^	0.84 ± 0.07^e^	1.24 ± 0.21^d^	0.97 ± 0.03^d^	1.0
Q2‐RY	0.53 ± 0.11^de^	0.72 ± 0.07^a^	0.73 ± 0.07^b^	0.79 ± 0.12^a^	0.74 ± 0.05^a^	1.12 ± 0.15^b^	0.87 ± 0.05^a^	0.9

Note

Mean ± *SD* (standard deviation); means in the same column with different superscripts are significantly different (*p* < .01) (*N* = 3). The reference pattern used to calculate the amino acid scores was as followed (mg/g protein): Thr‐40, Val‐50, Met + Cys‐35, Ile‐40, Leu‐70, Phe + Tyr‐60, Lys‐55.

The first limiting amino acids of two quinoa varieties were the sulfur amino acids, methionine and cysteine, with values ranging from 0.49 (Q1‐Q and Q2‐Q) to 0.56 (Q1‐MM). Threonine was the second limiting amino acid in the processed and raw quinoa samples, with values between 0.73 (Q2‐Q) and 0.83 (Q1‐HH). The scores of essential amino acids (EAASs) for the dehulled quinoa samples (Q1‐MM and Q2‐MM) were significantly higher than for the corresponding raw quinoa samples (Q1‐Q and Q2‐Q).The difference of the scores of cystine + methionine, threonine, isoleucine, leucine, tyrosine phenylalanine, and lysine of Q1‐MM and Q2‐MM were 14.29%, 3.9%, 5.43%, 3.49%, 1.49%, and 1.9% and 6.12%, 8.22% and 9.3%, 6.25%, 4.88%, and 0.99% compared to that of Q1‐Q and Q2‐Q, respectively. The difference in scores of the same essential amino acid between Q1‐MM and Q1‐Q was different from that between Q2‐MM and Q2‐Q; for example, the score of cystine + methionine in Q1‐MM was 14.29% higher than in Q1‐Q, while that in Q2‐Q was 6.12% less than in Q2‐MM, which was likely due to the differences in the varieties.

Baking resulted in an increase in the AAS values of cystine + methionine, threonine, isoleucine, and leucine, and the AAS of threonine, isoleucine, and leucine of the two quinoa varieties Q1‐HH and Q2‐HH was 7.79%, 5.43%, 5.81%, 6.85%, 9.30%, and 10% higher than that of Q1‐Q and Q2‐Q, respectively; yet, there were no difference in the scores of tyrosine phenylalanine and lysine between Q1‐HH and Q2‐HH, and Q1‐Q and Q2‐Q. The scores of valine, threonine, isoleucine, leucine, tyrosine + phenylalanine, and lysine of the extruded samples (Q1‐JY and Q2‐JY), the boiled samples (Q1‐ZZ and Q2‐ZZ), and the heated under pressure samples (Q1‐RY and Q2‐RY) were less than those of Q1‐Q and Q2‐Q, and heated treatment caused lower scores of leucine and lysine than those of the other essential amino acids in the heated processed quinoa samples. The results are consistent with the results found by Lisiewska, Kmiecik, and Słupski (2007); Słupski (2010) and Khattab, Arntfield, and Nyachoti (2009) also found increases or decreases in some amino acids in raw and cooked pulses depending on the type and origin of the studied pulses.

To evaluate the effect of boiling, extrusion, heated under pressure, and baking process on amino acids, the nutrient retention (AR) was calculated (Table 3). The AR of the total essential amino acids of the extruded samples (Q1‐JY and Q2‐JY) and the heated under pressure samples (Q1‐RY and Q2‐RY) were below 100%. In contrast, the AR of the dehulled samples, boiled samples, and baked samples was 100% or higher.

Overall, the results indicated how the different cooking methods affect the amino acid content and the protein quality of quinoa. With this information, it is possible to choose the best procedure to process quinoa in order to preserve or improve the protein quality.

### 3.4 The effect of processing on the oil content of quinoa

**FIGURE 1:**
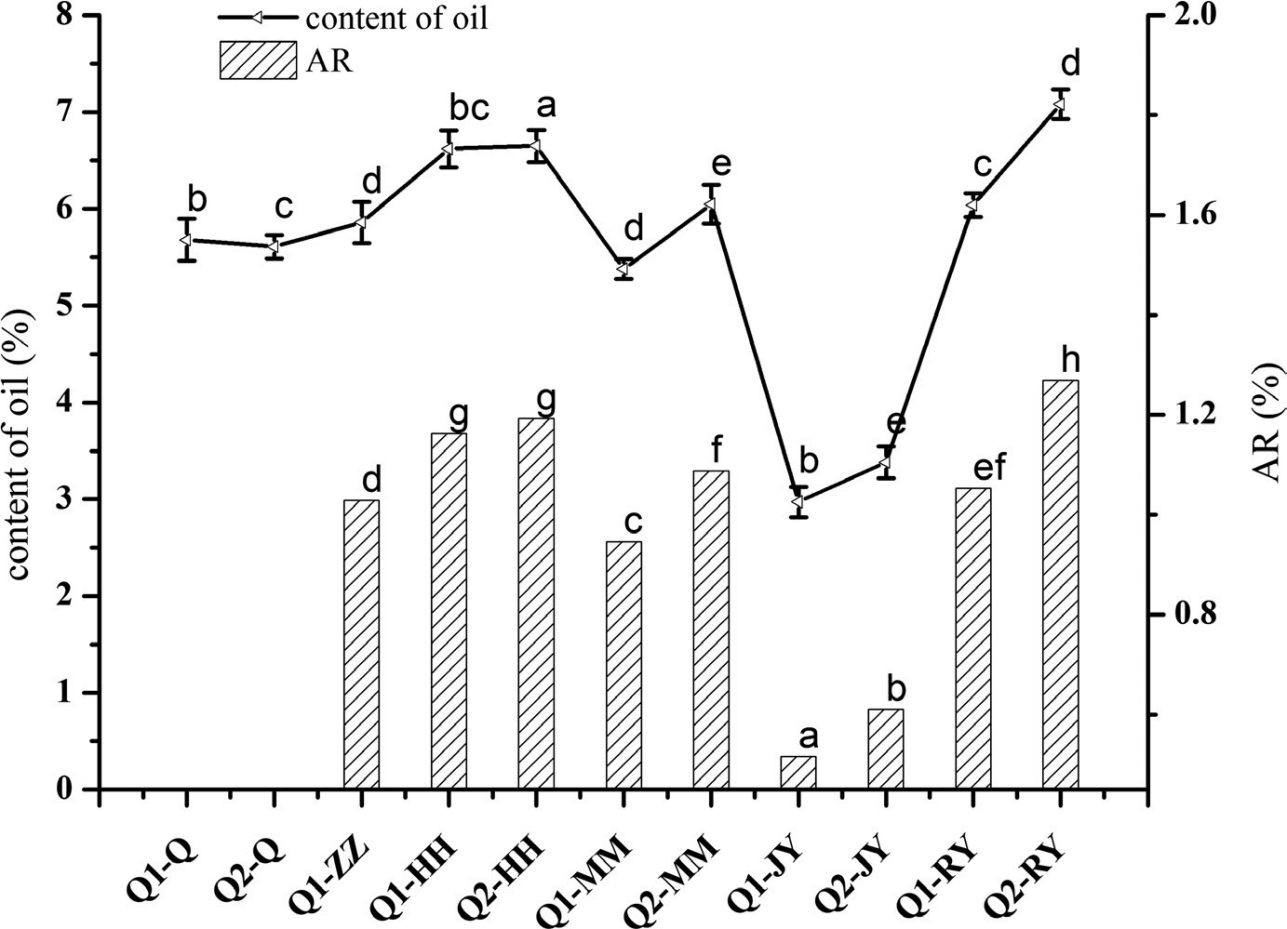
The effect of processing on the oil content and retention rate of quinoa. The different letter on curve of the oil content or column of AR of the quinoa of different processing methods indicates significant differences (*p* < .05)

The oil content of all samples is shown in Figure 1. The oil content of the raw quinoa samples Q1‐Q and Q2‐Q was 5.68% and 5.61%, respectively, and these results are consistent with the reports of Hager, Wolter, Jacob, Zannini, and Arendt (2012) and Dini, Rastrelli, Saturnino, and Schettino (1992). The oil content of Q1‐MM and Q2‐MM was 5.38% and 6.05%, respectively. There was a significant difference in the oil content between the control samples and the boiled, roasted, extruded, and heated under pressure samples, and the oil content of Q1‐ZZ, Q1‐HH, Q1‐RY, Q2‐ZZ, Q2‐HH, and Q2‐RY was 5.86%–7.20% higher than that of Q1‐Q, Q2‐Q Q1‐MM, and Q2‐MM; notably, the oil content of Q1‐RY and Q2‐RY was 7.20% and 7.08%, which was 26.76% and 26.20% higher than that of Q1‐Q and Q2‐Q, respectively.

The oil content of Q1‐JY and Q2‐JY was 2.97% and 3.38%, respectively, which was lower than that of Q1‐ZZ, Q1‐HH, Q1‐RY, Q2‐ZZ, Q2‐HH, and Q2‐RY, and was 47.71% and 39.75% lower than the oil content of the raw quinoa samples (Q1‐Q and Q2‐Q). The AR values of all samples except Q1‐JY and Q2‐JY were more than 1, and the loss of the oil in Q1‐JY and Q2‐JY was attributed to the impact of a high temperature and high pressure during the extrusion treatment. The dehulling, boiling, baking, and heating under pressure treatments may have preserved the oil in quinoa, and promote the possibility of utilization of oil in quinoa grains.

### 3.5 The effect of processing on the fatty acid composition of the oil in quinoa

The fatty acid composition of quinoa is shown in Table 4. The fatty acids in quinoa were mainly unsaturated fatty acids. The content of oleic acid, linoleic acid, and linolenic acid was 27%–34%, 43%–51.2%, and 6.9%–9.25%, respectively. The content linoleic acid was the highest in quinoa, while Peiretti, Gai, and Tassone (2013) reported the highest content of linolenic acid in quinoa grain, and the difference was probably due to varietal or geographical differences. The content of monounsaturated fatty acids was more than 30% of the total FA content.

**TABLE 4:** The fat acid composition of the oil in quinoa samples

	Sample	Palmitic acid	Palmitoleic acid	Octadecanoic acid	Oleinic acid	Linoleic acid	Linolenic acid	Arachic acid	Docosanoic acid	Erucidic acid
		C16:0	C16:1	C18:0	C18:1	C18:2	C18:3	C20:0	C22:0	C22:1
1	Q1‐Q	8.55 ± 0.31^a^	0.13 ± 0.04^d^	0.84 ± 0.01^ef^	29.58 ± 0.24^ef^	49.55 ± 0.71^d^	8.66 ± 0.33^f^	0.40 ± 0.01^a^	0.74 ± 0.04^f^	1.55 ± 0.05^bc^
2	Q1‐ZZ	8.75 ± 0.21^b^	0.09 ± 0.01^a^	0.86 ± 0.11^ef^	29.84 ± 0.32^f^	49.13 ± 0.68^b^	8.46 ± 0.53^c^	0.61 ± 0.002^e^	0.71 ± 0.05^d^	1.56 ± 0.07^c^
3	Q1‐HH	8.72 ± 0.15^b^	0.10 ± 0.04^ab^	0.86 ± 0.09^f^	29.45 ± 0.40^de^	49.58 ± 0.73^de^	8.62 ± 0.14^e^	0.40 ± 0.01^a^	0.73 ± 0.03^e^	1.56 ± 0.08^bc^
4	Q1‐MM	9.57 ± 0.31^e^	0.12 ± 0.08^cd^	0.95 ± 0.12^h^	30.28 ± 0.32^g^	48.89 ± 0.35^a^	6.90 ± 0.24^a^	0.69 ± 0.01^e^	0.86 ± 0.05^i^	1.74 ± 0.04^f^
5	Q1‐JY	8.84 ± 0.11^b^	0.20 ± 0.09^h^	0.93 ± 0.01^g^	29.13 ± 0.40^d^	49.98 ± 0.22^f^	8.30 ± 0.33^b^	0.42 ± 0.05^ab^	0.79 ± 0.04^h^	1.42 ± 0.06^a^
6	Q1‐RY	8.66 ± 0.24^b^	0.16 ± 0.08^g^	0.83 ± 0.13^d^	29.35 ± 0.35^de^	49.69 ± 0.23^e^	8.68 ± 0.12^f^	0.39 ± 0.04^a^	0.69 ± 0.03^c^	1.56 ± 0.08^bc^
7	Q2‐Q	9.07 ± 0.20^c^	0.15 ± 0.06^ef^	0.70 ± 0.07^a^	27.03 ± 0.31^bc^	51.26 ± 0.93^hi^	9.25 ± 0.04^j^	0.36 ± 0.02^a^	0.67 ± 0.05^b^	1.52 ± 0.05^b^
8	Q2‐ZZ	9.42 ± 0.15^ab^	0.14 ± 0.07^e^	0.84 ± 0.08^e^	29.52 ± 0.27^de^	49.38 ± 0.70^c^	8.51 ± 0.10^d^	0.51 ± 0.07^c^	0.76 ± 0.05^g^	1.63 ± 0.06^e^
9	Q2‐HH	9.12 ± 0.14^c^	0.13 ± 0.04^d^	0.69 ± 0.09^a^	26.89 ± 0.32^ab^	51.20 ± 0.73^h^	9.19 ± 0.22^i^	0.50 ± 0.05^bc^	0.69 ± 0.05^c^	1.60 ± 0.04^de^
10	Q2‐MM	9.06 ± 0.14^c^	0.11 ± 0.07^bc^	0.69 ± 0.13^a^	28.35 ± 0.35^cd^	50.69 ± 0.23^g^	8.680 ± 0.12^f^	0.33 ± 0.04^a^	0.63 ± 0.04^a^	1.57 ± 0.05^cd^
11	Q2‐JY	9.38 ± 0.22^d^	0.15 ± 0.07^fg^	0.75 ± 0.04^b^	26.67 ± 0.41^a^	51.45 ± 0.24^i^	8.95 ± 0.41^g^	0.37 ± 0.05^a^	0.74 ± 0.06^f^	1.54 ± 0.07^bc^
12	Q2‐RY	9.41 ± 0.10^d^	0.15 ± 0.06^ef^	0.77 ± 0.07^c^	27.21 ± 0.38^c^	50.75 ± 0.87^g^	9.08 ± 0.20^h^	0.36 ± 0.06^a^	0.67 ± 0.03^b^	1.60 ± 0.08^de^

Note

Mean ± *SD* (standard deviation); means in the same column with different superscripts are significantly different (*p* < .01) (*N* = 3).

The effects of different processing treatment on the composition of quinoa fatty acids were different, and the effects of processing on different fatty acids were different. The percentage of linolenic acid in the total fatty acid content of dehulled, boiled, baked, extruded, and heated under pressure samples were less than that in the raw quinoa samples (*p* < .01), and the dehulled quinoa had the lowest proportion of linolenic acid in both quinoa varieties (Q1‐MM and Q2‐MM). The proportion of linolenic acid in Q1‐MM and Q2‐MM was 20% and 6% lower than that of Q1‐Q and Q2‐Q, respectively (*p* < .01). The proportion of palmitoleic acid in the boiled, baked, and dehulled samples was significant lower than in samples Q1‐Q and Q2‐Q (*p* < .01), and the proportion of palmitoleic acid in Q1‐ZZ and Q2‐ZZ was 74% and 77% and that in Q1‐HH and Q2‐HH was 80% and 84% than that in Q1‐1 and Q2‐Q, respectively. The proportion of fatty acids in the extruded and heated under pressure samples was close to that in the raw quinoa samples.

### 3.6 The effect of processing on the hydrolysis of starch in quinoa

**FIGURE 2:**
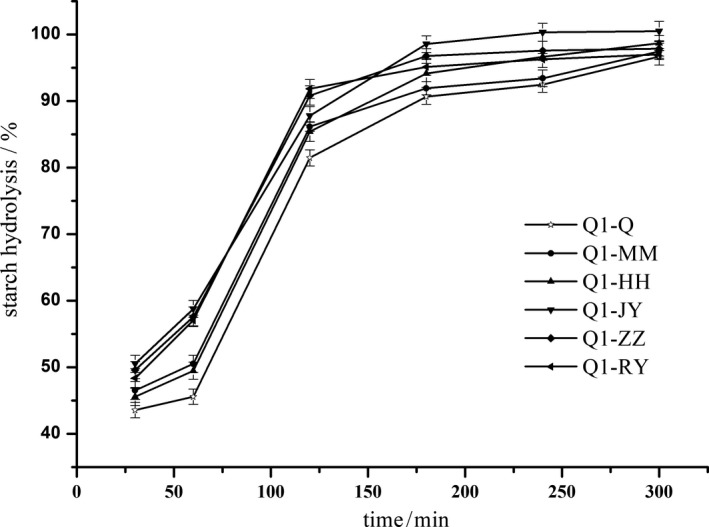
The effect of processing on hydrolysis of starch in quinoa (Q1 cultivated varieties). Q1‐Q—raw quinoa of cultivated varieties Q1; Q1‐MM—dehulled quinoa of cultivated varieties Q1; Q1‐HH—baked quinoa cultivated varieties Q1; Q1‐JY—extrusion quinoa cultivated varieties Q1; Q1‐ZZ—boiled quinoa cultivated varieties Q1; Q1‐RY—heated under pressure quinoa cultivated varieties Q1

**FIGURE 3:**
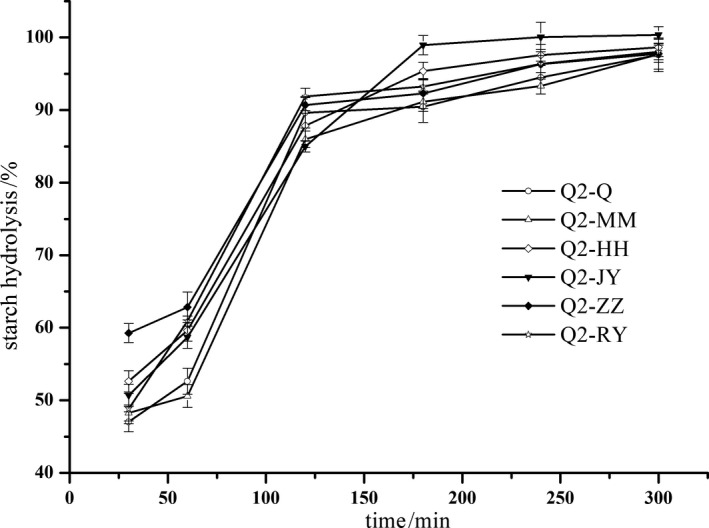
The effect of processing on hydrolysis of starch in quinoa (Q2 cultivated varieties). Q2‐Q—raw quinoa of cultivated varieties Q2; Q2‐MM—dehulled quinoa of cultivated varieties Q2; Q2‐HH—baked quinoa cultivated varieties Q2; Q2‐JY—extrusion quinoa cultivated varieties Q2; Q2‐ZZ—boiled quinoa cultivated varieties Q2; Q2‐RY—heated under pressure quinoa cultivated varieties Q2

The effect of processing on the hydrolysis rate of starch in quinoa is shown in Figures 2 and 3. The hydrolyzed samples were extracted for 30, 60, 120, 180, 240, and 300 min, respectively. The glucose content was determined, and the hydrolysis percentage of starch was expressed as a percentage of the glucose content relative to the initial weight of starch in the quinoa sample. The hydrolysis rate of all samples increased rapidly from 30 to 120 min and then slowly increased after 120 min, but the increase rates of all samples (Q1‐Q, Q2‐Q, Q1‐MM, Q2‐MM, Q1‐ZZ, Q2‐ZZ, Q1‐HH, Q2‐HH, Q1‐JY, Q2‐JY, Q1‐RY, and Q2‐RY) were different. The starch hydrolysis rate of the raw quinoa samples (Q1‐Q and Q2‐Q) was lower than that of processed samples at each sampling point, and the hydrolysis rate plateaued on the curve of hydrolysis versus time after 120 min of hydrolysis. In addition, the samples were not completely hydrolyzed even after 300 min, which was consistent with results reported by Srichuwong et al. (2017), but the hydrolysis rate at 120 min was lower than that reported by Srichuwong et al. (2017). This may be due to the different quinoa varieties and to several factors in quinoa samples, including physically damaged starch granules, proteins, lipids, and phytochemicals that may have an additional impact on enzyme substrate accessibility and available enzyme activities. Hydrolysis may release phenolic compounds and phytic acid from quinoa, which could inhibit α‐amylase activity and reduce the rate of starch digestion at certain concentrations (Deshpande & Cheryan, 1984; Thompson & Yoon, 1984). Consequently, the enzymatic hydrolysis of starch in quinoa resulted from the interplay of several factors, including starch structures, protein matrices, enzyme inhibitors, and phytochemicals, which would be further varied with the degree and method of thermal treatment.

Ayyash et al. (2019) reported that the inhibitory effect of quinoa whole grain powder on glucosidase was 30%–60%, and the mechanism of inhibition on glucosidase was attributed to peptides and small molecular peptides (McCue, Kwon, & Shetty, 2005). However, the hydrolysis rate of Q1‐MM and Q2‐MM was significantly higher than that of raw quinoa, while dehulling the quinoa slightly scoured the outer layer of the pericarp; therefore, the factors leading to the low hydrolysis rate of quinoa grain may be owed to peptides and small peptides inhibiting amylase activity. The reason needs to be further studied and discussed. The low hydrolysis rate of quinoa reduces the glucose release rate from quinoa caused by digesting effective carbohydrates, and this reduces the risk of a rapid increase in blood glucose level, helping control type 2 diabetes mellitus (Yu, Yin, Zhao, Liu, & Chen, 2012).

## 4 CONCLUSION

Although 8.6% of the quinoa mass was removed during dehulling, the recovery rate of total essential amino acids was 100% in the dehulled quinoa samples; the hydrolysis degree of the dehulled quinoa samples was higher than that the raw samples, which indicated that the essential amino acids, fat, and starch of quinoa could be fully utilized after moderate dehulling. Extrusion is a versatile food manufacturing technique that offers flexible processing of food with diverse textures and shapes, and can be used to manufacture a variety of ready‐to‐eat casual quinoa food with a crisp texture and the variable size, but it was found that the loss of fat content caused by extrusion treatment of quinoa was very large in this paper, and the loss of essential amino acids should also be noted. Compared with the processes of extrusion, baking, and heating under pressure processing treatments, boiling the quinoa exhibited a high recovery rate of the total essential amino acids and fatty acids, which may be used as an optimal heated processing technique for quinoa. The proper processing methods for quinoa products to satisfy the nutritional demand of consumers are important. The results of this paper may help commercial or production industries easily choose the appropriate processing method to meet the consumer's request for nutrition in quinoa grain, effectively increase the value of quinoa products, and enhance its industrial profits.

## CONFLICT OF INTEREST

There are no conflicts of interest.

## ETHICAL APPROVAL

This study does not involve any human or animal testing.
